# Boosting Genomic Prediction Transferability with Sparse Testing

**DOI:** 10.3390/genes16070827

**Published:** 2025-07-16

**Authors:** Osval A. Montesinos-López, Jose Crossa, Paolo Vitale, Guillermo Gerard, Leonardo Crespo-Herrera, Susanne Dreisigacker, Carolina Saint Pierre, Iván Delgado-Enciso, Abelardo Montesinos-López, Reka Howard

**Affiliations:** 1 Facultad de Telemática, Universidad de Colima, Colima 28040, Col., Mexico; osval78t@gmail.com; 2 International Maize and Wheat Improvement Center (CIMMYT), Km 45, Carretera Mexico-Veracruz, Texcoco 52640, Edo. Mex., Mexico; j.crossa@cgiar.org (J.C.); p.vitale@cgiar.org (P.V.); g.gerard@cgiar.org (G.G.); l.crespo@cgiar.org (L.C.-H.); s.dreisigacker@cgiar.org (S.D.); c.saintpierre@cgiar.org (C.S.P.); 3 Colegio de Postgraduados, Montecillos, Texcoco 56230, Edo. Mex., Mexico; 4 School of Medicine, University of Colima, Colima 28040, Col., Mexico; ivan_delgado_enciso@ucol.mx; 5 Centro Universitario de Ciencias Eactas e Ingenierías (CUCEI), Universidad de Guadalajara, Guadalajara 44430, Jal., Mexico; 6 Department of Statistics, University of Nebraska-Lincoln, 343C Hardin Hall, Lincoln, NE 68583-0963, USA

**Keywords:** sparse testing, tested lines in untested environment, genomic prediction

## Abstract

Background/Objectives: Improving sparse testing is essential for enhancing the efficiency of genomic prediction (GP). Accordingly, new strategies are being explored to refine genomic selection (GS) methods under sparse testing conditions. Methods: In this study, a sparse testing approach was evaluated, specifically in the context of predicting performance for tested lines in untested environments. Sparse testing is particularly practical in large-scale breeding programs because it reduces the cost and logistical burden of evaluating every genotype in every environment, while still enabling accurate prediction through strategic data use. To achieve this, we used training data from CIMMYT (Obregon, Mexico), along with partial data from India, to predict line performance in India using observations from Mexico. Results: Our results show that incorporating data from Obregon into the training set improved prediction accuracy, with greater effectiveness when the data were temporally closer. Across environments, Pearson’s correlation improved by at least 219% (in a testing proportion of 50%), while gains in the percentage of matching in top 10% and 20% of top lines were 18.42% and 20.79%, respectively (also in a testing proportion of 50%). Conclusions: These findings emphasize that enriching training data with relevant, temporally proximate information is key to enhancing genomic prediction performance; conversely, incorporating unrelated data can reduce prediction accuracy.

## 1. Introduction

Genomic prediction (GP) is transforming plant breeding by enabling scientists to identify high-performing genetic profiles earlier in the breeding process, significantly reducing the time and costs associated with developing improved crop varieties. Unlike traditional breeding, which relies heavily on observable traits and lengthy field trials, GP leverages genomic data to predict plant performance, even for complex traits like yield stability and disease resistance. By integrating vast amounts of genetic information with machine learning algorithms, GP allows breeders to make faster and more accurate selection decisions, improving both the precision and efficiency of breeding programs. As a result, it is now possible to breed plants that are better adapted to specific climates and stresses, supporting food security and resilience against climate change worldwide. This shift towards data-driven selection is helping to sustain agricultural productivity in the face of environmental challenges, ultimately benefiting both breeders and farmers globally [1,2].

Implementing genomic prediction in plant breeding remains challenging due to complex genetic and statistical factors. One significant hurdle is the high dimensionality of genomic data, where the number of markers often exceeds the sample size, creating multicollinearity issues. This complexity demands sophisticated statistical models that can handle these data intricacies, especially for polygenic traits controlled by numerous small-effect loci. Additionally, genotype-by-environment (G × E) interactions complicate predictions, as the performance of genotypes can vary widely across environments. Accounting for these interactions requires advanced models to capture genetic correlations across diverse environments, which increases computational demands. Another challenge is the high cost of genotyping large populations, especially in developing countries where resources may be limited, further slowing the adoption of genomic selection technologies [1,3,4].

For this reason, many strategies have been implemented in GP with the goal of improving its efficiency. One of these strategies is called sparse testing. Sparse testing is crucial in genomic prediction as it enables the evaluation of a wide variety of cultivars across multiple environments without the cost and logistical constraints of fully testing each of them in every environment. By strategically selecting and testing only a subset of genotypes in specific environments, sparse testing helps generate sufficient data to build accurate prediction models that account for G × E, allowing breeders to predict untested combinations effectively. This approach is particularly beneficial in large-scale breeding programs, where it reduces field trial costs and resource demands while maintaining the prediction power required for selecting cultivars suited to varied environmental conditions. Moreover, sparse testing supports data efficiency, enhancing the ability to predict performance in unobserved environments, ultimately accelerating the breeding cycle and improving genetic gains across diverse climates [1,5].

Recent developments in machine learning have led to the integration of non-linear and deep learning models into genomic prediction, offering the potential to capture complex trait architectures and G × E interactions more effectively than traditional linear methods. Models such as convolutional neural networks (CNNs), multilayer perceptrons (MLPs), and hybrid ensemble frameworks have demonstrated competitive performance, especially when dealing with high-dimensional genomic and environmental data [6]. While these models offer advantages in flexibility and potential accuracy, they also require large datasets and careful tuning, which may not always be feasible in breeding contexts with limited training data. Thus, GBLUP remains a robust and widely used benchmark model for evaluating genomic prediction strategies, including those involving sparse testing.

In plant breeding, multi-environment trials (METs) are critical for accurately evaluating genotype performance and stability under diverse environmental conditions. Genomic prediction (GP) models that incorporate genotype-by-environment (G × E) interactions have significantly advanced breeding programs by predicting the performance of unobserved genotype–environment combinations. In crop improvement, many cultivars (varieties) (called genotypes) have been observed in different places or years (called environments). Breeders have data from those varieties in some environments, but not in others, and we must predict how those same varieties would do in the missing environments. So, breeders train the model using the observed environments and then test the model by predicting the performance in the environments where varieties were not observed. The cross-validation (CV2-type cross-validation scheme), initially introduced by Burgueño et al. (2012) [7], specifically addresses realistic scenarios encountered in plant breeding programs where some genotype–environment combinations are deliberately masked, simulating situations where genotypes have incomplete environmental testing due to resource limitations or logistical constraints. This approach allows for a realistic assessment of genomic prediction models’ capability to estimate genotype performance in environments where no direct phenotypic data exist.

Since its initial proposal, the CV2 methodology has evolved to reflect practical constraints and opportunities within breeding programs. For example, Montesinos et al. (2024) [8] integrated sparse testing methodologies, applying incomplete block and random allocation designs to further simulate realistic breeding scenarios. Additionally, this study further expanded upon the CV2 concept by strategically enriching training datasets with related environmental data, aiming to enhance predictive accuracy in untested environments. These advancements illustrate the versatility and adaptability of CV2-based strategies within modern genomic selection practices.

In this research, we will explore sparse testing for tested lines in untested environments. This type of sparse testing allows breeders to predict the performance of tested genotypes in untested environments by leveraging information from strategically tested lines in various conditions. This approach helps to identify robust genotypes capable of thriving across different environments, even when complete testing in all conditions is impractical. Sparse testing frameworks rely on statistical and genomic models that use data from tested genotypes to infer the potential of similar but untested genotypes, addressing GE with fewer resources. By optimizing the selection of test sites and genotypes, sparse testing improves efficiency, reducing costs and labor while maintaining high predictive accuracy. This method is particularly advantageous in large-scale breeding programs with limited testing budgets and in regions with diverse and variable climates, where anticipating genotype adaptation is essential [1,5,7].

In this study, we assess the predictive capacity of sparse testing under tested lines in untested environments using a real-world dataset from South Asian Target Population of Environments (TPEs), encompassing 25 unique site–year combinations. Our analysis simulates scenarios where specific genotypes are evaluated in certain environments but are absent in others. These approaches include methods for predicting missing lines for a specific environment using information on other environments with related lines.

This work builds upon our previous study [8], which evaluated sparse testing under random and incomplete block designs. Here, we focus on a more realistic and operationally relevant sparse testing scenario—predicting tested lines in untested environments—while leveraging multi-year, multi-environmental data enrichment. By explicitly comparing enriched versus non-enriched training sets, this study adds new insights into the transferability of genomic predictions under practical field conditions.

## 2. Materials and Methods

### 2.1. Datasets

The experimental material comprised 941 elite wheat lines from CIMMYT (Table 1). These genotypes were evaluated for grain yield (GY) over two consecutive crop seasons across three target population environments (TPEs). Of the total wheat lines, 444 were tested in the 2021–2022 growing season, with the remaining 497 were evaluated in the 2022–2023 season. In the 2021–2022 season, 166 lines were assigned to TPE1 (4 locations in India and 3 locations in Obregon, México), 165 to TPE2 (5 locations in India and 3 locations in Obregon, México), and 112 to TPE3 (2 locations in India and 3 locations in Obregon, México). In the 2022–2023 season, 166 genotypes were planted in each TPE: TPE1 (6 locations in India and 6 in Obregon, México), TPE2 (6 locations in India and 6 in Obregon, México), and TPE3 (3 locations in India and 6 in Obregon, México). At each location, an alpha lattice design with two replications was established to optimize cost efficiency while ensuring robust parameter estimation, yielding reliable results for CIMMYT’s breeding programs.

#### Description of the Target Population of Environments (TPEs)

In Mexico, all evaluations were conducted at CENEB (Centro Experimental Norman E. Borlaug) in Ciudad Obregón, Sonora (27.4936° N, 109.9380° W), under fully irrigated conditions typical of the northwestern wheat belt. Obregón has a median maximum daily temperature of 32 °C during the growing season, with total seasonal rainfall below 50 mm, necessitating full irrigation. Soils are predominantly clay loam with high fertility, and trials are managed with high-input protocols.

In India, trials were carried out at representative sites of the All India Coordinated Wheat Improvement Program (AICWIP), including the following: Ludhiana (30.9010° N, 75.8573° E)—northwest plains; timely sown, moderate rainfall (300–400 mm), clay loam soils. Pusa (25.9852° N, 85.6638° E)—Eastern Indo-Gangetic plains; warmer, sub-tropical climate with annual rainfall ~1000 mm, sandy loam soils. Wellington (11.3724° N, 76.7850° E)—southern hills; temperate climate with high humidity (~70–90%), cooler night temperatures, and well-drained forest soils.

Regarding the genetic material, all evaluated wheat lines were elite breeding lines from CIMMYT’s spring wheat program. A total of 941 unique genotypes were included in the study, with subsets planted across TPEs. In each TPE × year combination, distinct but partially overlapping subsets of genotypes were evaluated. For example, 166 lines were planted in TPE1 in 2021–2022 and another 166 in 2022–2023. Some genotypes were shared across years and sites to enable sparse testing designs.

Environments were grouped into TPEs using expert knowledge of breeding programs and the clustering of historical yield and environmental covariates (e.g., temperature, rainfall). This TPE classification allows us to evaluate the potential for sparse testing, where only a subset of lines is evaluated in a subset of sites within each TPE, and genomic prediction is used to infer performance in untested environments within the same TPE. This approach is consistent with the operational needs of large-scale breeding programs in both countries.

It is important to highlight that the same lines under study in each dataset were evaluated across all environments in both countries (India and Mexico). In Mexico, all evaluations were conducted in Cd. Obregon, Sonora, while in India, they were carried out in Ludhiana. This consistent evaluation approach within each country ensures the comparability of results across environments and strengthens the reliability of genotype performance assessments.

### 2.2. Bayesian GBLUP Model

The multi-environment GBLUP model implemented:

(1) $$Y_{ij}=\mu +E_{i}+g_{j}+gE_{ij}+\epsilon_{ij}$$

where $Y_{ij}$ represents the Best Linear Unbiased Estimate (BLUE) for the i-th genotype in the j-th environment. The grand mean is denoted by μ, and the random effects associated with environments, $E_{i}$ for i = 1,…,I, are assumed to follow a multivariate normal distribution $E={\left(E_{1},\ldots ,E_{I}\right)}^{T}∼N_{J}\left(0,\sigma_{E}^{2}I_{E}\right)$, where $I_{E}$ is the identity covariance matrix of environments, and $\sigma_{E}^{2}$ represents the variance component attributed to environmental effects. Additionally, $g_{j},$ j=1,…,J, are the random effects of genotypes (lines), and $gE_{ij}$ denotes the random effects associated with the genotype-by-environment interaction. The residual errors, $\epsilon_{ij}$, are assumed to be independent and normally distributed with mean 0 and variance $\sigma^{2}$. Furthermore, the genotypic random effects vector $g={\left(g_{1},\ldots ,g_{J}\right)}^{T}∼N_{J}\left(0,\sigma_{g}^{2}G\right)$, where G is the genomic relationship matrix [9], and $\sigma_{g}^{2}$ is the genetic variance component. The genotype-by-environment interaction effects, $gE={\left(gE_{11},\ldots ,gE_{1J},\ldots ,\;gE_{IJ}\right)}^{T}$, are modeled as following a multivariate normal distribution $gE∼N_{IJ}\left(0,\sigma_{gE}^{2}\left(Z_{g}GZ_{g}^{T}{}^{\circ}Z_{E}I_{E}Z_{E}^{T}\right)\right)$, where $Z_{g}$ is the incidence matrix for the additive genetic effects, the variance component $\sigma_{gE}^{2}$ corresponds to the genotype-by-environment interaction, ° denotes the Hadamard product, $Z_{E}\;$ is the incidence matrix representing the environmental effects, and $I_{E}$ is the identity matrix denoting independent environments. The implementation of this model was carried out using the BGLR package [10]. Finally, the residual error components $\epsilon_{ij}$ were assumed to be distributed as $\epsilon_{ij}∼N_{J}\left(0,\sigma_{\epsilon }^{2}\right)$, where $\sigma_{\epsilon }^{2}$ is the error variance.

#### Why Using GBLUP and GBLUP_Ad?

In this study, we focused on the genomic best linear unbiased predictor (GBLUP) and its enriched variant (GBLUP_Ad) to isolate and evaluate the effects of training data composition under sparse testing conditions. While more complex models such as reproducing kernel Hilbert space (RKHS) regression, Bayesian Lasso, and deep learning approaches have been successfully applied in genomic prediction, our aim was not to compare predictive algorithms but to assess how strategic data enrichment can improve prediction accuracy in untested environments. GBLUP was selected for its widespread use, ease of implementation, and ability to provide a stable reference point for evaluating the impact of cross-environment training scenarios. Future work may incorporate non-linear models to further investigate whether they can better capture G × E interactions under similar sparse testing settings.

### 2.3. Cross-Validation Schemes

Two primary cross-validation strategies were employed to evaluate the prediction accuracy of sparse testing approaches.

#### 2.3.1. Cross-Validation Strategy 1

A 10-fold random partitioning scheme was used for all target environments in India. The training data consisted of 85%, 70%, 50%, and 30% of the lines, while the remaining 15%, 30%, 50%, and 70%, respectively, were reserved for testing (target population). The results from this strategy, using only data from the target environment in India, were denoted as GBLUP.

#### 2.3.2. Cross-Validation Strategy 2 (Incorporating Additional Training Data to TARGET Data)

This strategy enhanced the training set by including data from previous years in India, along with data from Obregon, Sonora, Mexico (both from the current and previous years, when available). This approach was labeled GBLUP_Ad, emphasizing the impact of enriched, multi-environmental training datasets on model performance.

For instance, when the testing set consisted of 15%, 30%, 50%, and 70% of the lines from India in the target environment TPE_3_2022_2023, the training set comprised the following:

- The remaining 85%, 70%, 50%, and 30% of lines from India in TPE_3_2022_2023.
- All lines from India in TPE_3_2021_2022.
- All lines from Obregon, Sonora, Mexico, from both TPE_3_2021_2022 and TPE_3_2022_2023.

### 2.4. Model Performance Evaluation and Comparisons

Model performance was evaluated using two key metrics: (1) Average Pearson’s correlation (COR), that is a measure of the linear correlation between observed and predicted values across 10 partitions, and (2) Percentage of Matching in the top-performing lines, which includes the percentage of overlap between observed and predicted lines in the top 10% (PM_10) and top 20% (PM_20) of performance. Collectively, these metrics provided a comprehensive assessment of prediction accuracy across all random partitions.

Although statistical tests such as paired *t*-tests or confidence intervals are widely used in other contexts, they are not appropriate for comparing model performance within standard k-fold cross-validation frameworks. This is because the cross-validation folds are not independent: the training and testing partitions typically overlap, violating the assumption of independent and identically distributed samples required for valid statistical inference. As demonstrated by [11], there exists no unbiased estimator of the variance in k-fold cross-validation, and any attempt to estimate significance based on such partitions may lead to incorrect conclusions. Similarly, [12] highlighted that performing model selection and evaluation within the same cross-validation framework can introduce bias and artificially inflate significance. For this reason, we follow established best practices in genomic prediction by reporting the average prediction metrics (e.g., Pearson’s correlation, PM_10, PM_20), along with their standard deviations and standard errors across folds, which offer a more robust and interpretable measure of model performance.

## 3. Results

The results are presented in four sections. Section 3.1, Section 3.2, Section 3.3 and Section 3.4 contain the results for the datasets TPE_1_2021_2022, TPE_2_2021_2022, and TPE_3_2022_2023 and across, respectively. Meanwhile, Section 3.4 provides the results across all datasets (Across data). Finally, Appendix B and Appendix C provide the figures and tables corresponding to the datasets TPE_1_2022_2023, TPE_2_2022_2023, and TPE_3_2021_2022. The results are presented in terms of three metrics: the Pearson’s Correlation (COR), Percentage of Matching in the top 10% (PM_10), and Percentage of Matching in the top 20% (PM_20) for each dataset.

In some scenarios, the baseline GBLUP model produced negative Pearson’s correlation values or extreme relative efficiency (RE) scores. These negative values reflect instances where the model failed to generalize to the testing set, often due to limited or uninformative training data. The RE metric was calculated as the percentage change in the squared correlation of GBLUP relative to GBLUP_Ad, which can result in large or undefined values when the baseline model’s correlation approaches zero or becomes negative. While such values may seem extreme, they are useful in highlighting the extent to which GBLUP_Ad improves prediction under sparse or biologically dissimilar training conditions. Importantly, these results also emphasize the need to carefully interpret low or negative correlations as signals of limited transferability between training and testing environments.

### 3.1. TPE_1_2021_2022

**Figure 1:**
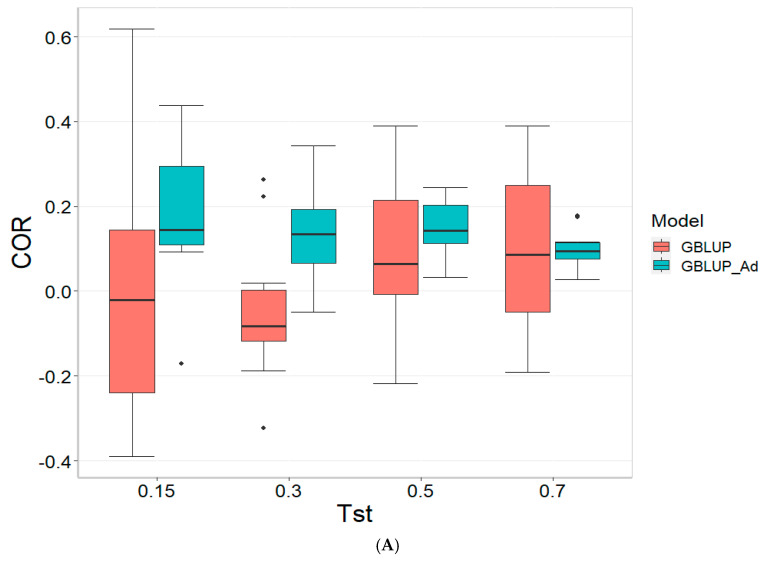

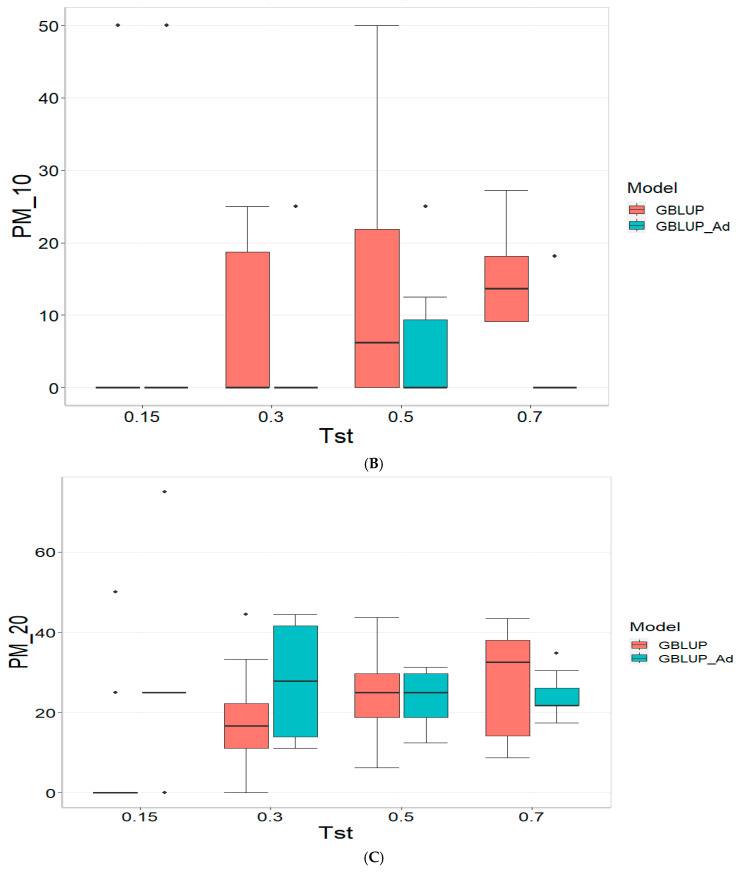
Comparative performance of genomic prediction models in terms of Pearson correlation (COR) (**A**), percentage of agreement in the top 10% (PM_10) (**B**) and top 20% (PM_20) (**C**) for TPE_1_2021_2022, using random cross-validation. Tst denotes the proportion of testing set. For each metric (COR, PM_10, PM_20), standard errors were calculated across the 10 cross-validation folds. These error bars provide an estimate of variability and aid in the interpretation of model stability across replicates.

Figure 1 presents the results for the dataset TPE_1_2021_2022 under a comparative analysis of the models GBLUP and GBLUP_Ad in terms of their predictive efficiency, measured by Pearson’s correlation (COR), and the Percentage of Matching for the selected optimal lines in the top 10% and 20% (PM_10 and PM_20). For further details, please refer to Table A1 in Appendix A.

In the analysis, the GBLUP_Ad model demonstrates superior performance across all evaluated metrics (COR, PM_10, PM_20) compared to GBLUP for several scenarios, especially for COR. For the COR metric, GBLUP_Ad maintains positive averages, with means ranging from 0.101 to 0.179 across different Tst values (where Tst denotes the proportion of testing set with possible values of 0.15, 0.30, 0.50, and 0.70), while GBLUP shows negative averages for the lower Tst values, such as −0.017 for Tst = 0.15 and −0.045 for TST = 0.30, reflecting its lower performance.

Regarding the PM_10 and PM_20 metrics, GBLUP_Ad outperforms GBLUP for some cases. For Tst = 0.15 and PM_20, the mean value for GBLUP_Ad is 25.000 compared to 7.500 for GBLUP. Also, for Tst = 0.30 and PM_20, the mean is 27.778 for GBLUP_Ad compared for GBLUP having a mean of 17.778. For the other scenarios comparing the metrics PM_10 and PM_20, GBLUP outperforms GBLUP_Ad in terms of the mean.

Overall, the relative efficiency of GBLUP is negative or significantly lower, whereas GBLUP_Ad establishes itself as the reference model with a relative efficiency of 0%, consolidating its superiority in all evaluated aspects.

### 3.2. TPE_2_2021_2022

**Figure 2:**
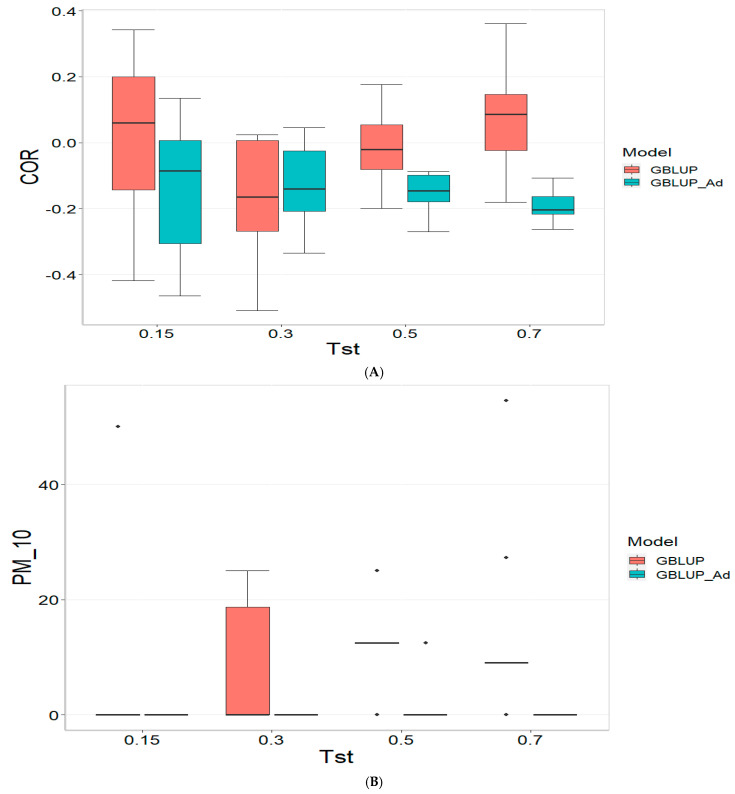

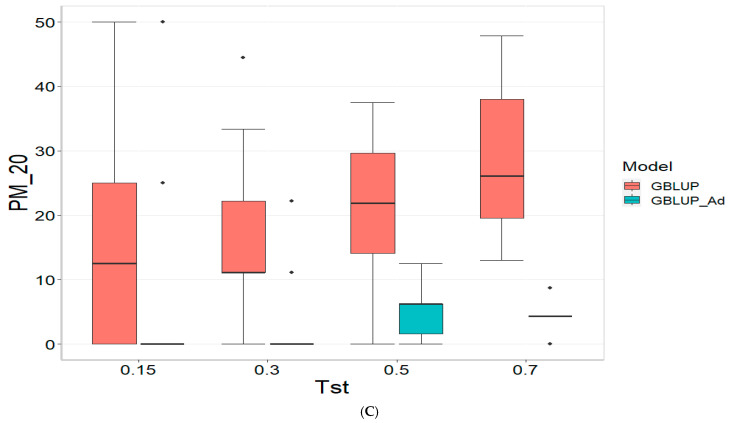
Comparative performance of genomic prediction models in terms of Pearson correlation (COR) (**A**), and percentage of agreement in the top 10% (PM_10) (**B**) and top 20% (PM_20) (**C**) for TPE_2_2021_2022, using random cross-validation. Tst denotes the proportion of testing set. For each metric (COR, PM_10, PM_20), standard errors were calculated across the 10 cross-validation folds. These error bars provide an estimate of variability and aid in the interpretation of model stability across replicates.

Figure 2 presents the results for TPE_2_2021_2022 under a comparative analysis of the GBLUP and GBLUP_Ad models in terms of COR, PM_10 and PM_20. For further details, please refer to Table A2 in Appendix A.

For the COR metric, GBLUP shows better performance at Tst = 0.15 and Tst = 0.70, with averages of 0.024 and 0.081, respectively, while GBLUP_Ad presents negative averages across all evaluated Tst, ranging from −0.148 to −0.194. However, the standard deviation of GBLUP_Ad is generally lower, suggesting more consistent predictions, although with overall lower performance. The relative efficiency (RE) of GBLUP is negative at Tst = 0.15 and Tst = 0.70, indicating inferior performance compared to GBLUP_Ad.

For the PM_10 metric, GBLUP_Ad shows little variability in the early Tst, with averages of 0.000 at several points, while GBLUP has higher averages, such as 13.636 at TST = 0.70. However, the relative efficiency of GBLUP is negative or low across all Tst, reinforcing the superiority of GBLUP_Ad in terms of efficiency and accuracy. Finally, for the PM_20 metric, GBLUP_Ad has lower averages and smaller standard deviations compared to GBLUP, which has averages like 28.696 for Tst = 0.70. The relative efficiency of GBLUP is negative in most cases, while GBLUP_Ad demonstrates greater consistency and efficiency.

Although GBLUP shows some positive average values in certain metrics and Tst, GBLUP_Ad excels in terms of consistency and lower variability, making it generally more efficient, as reflected by the low or zero relative efficiency rates compared to GBLUP.

### 3.3. TPE_3_2022_2023

**Figure 3:**
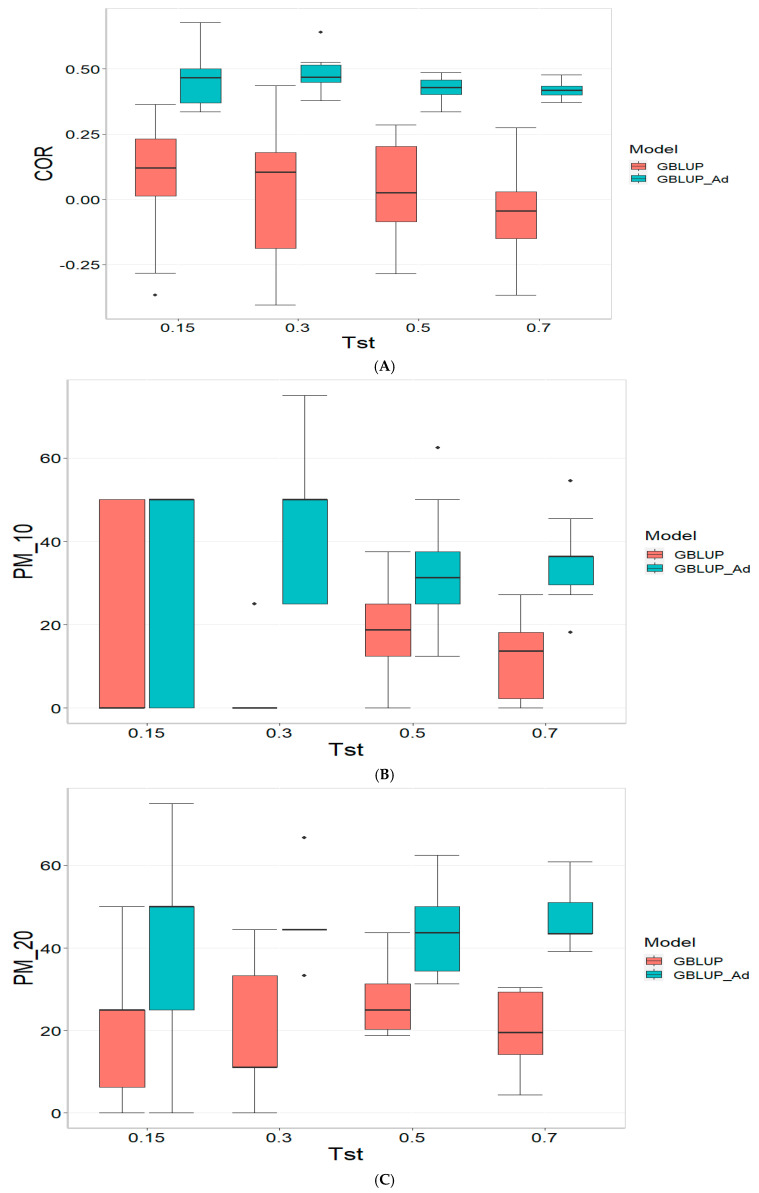
Comparative performance of genomic prediction models in terms of Pearson correlation (COR) (**A**), and percentage of agreement in the top 10% (PM_10) (**B**) and top 20% (PM_20) (**C**) for TPE_3_2022_2023, using random cross-validation. Tst denotes the proportion of testing set. For each metric (COR, PM_10, PM_20), standard errors were calculated across the 10 cross-validation folds. These error bars provide an estimate of variability and aid in the interpretation of model stability across replicates.

The results for the TPE_3_2022_2023 dataset are presented in Figure 3. For more details, please refer to Table A3 in Appendix A.

For the COR metric, for Tst = 0.15, the GBLUP_Ad model demonstrates superior performance with a mean value of 0.455 and a low standard deviation of 0.104, suggesting more consistent and accurate predictions. In contrast, GBLUP has a mean value of 0.073 and a higher standard deviation of 0.236, indicating lower accuracy. The relative efficiency (RE) of GBLUP is high, suggesting inferior performance compared to GBLUP_Ad. As Tst increases, GBLUP_Ad continues to outperform GBLUP. For example, at Tst = 0.70, GBLUP_Ad shows a mean of 0.418 and a standard deviation of 0.029, while GBLUP shows a negative mean of −0.029 and a standard deviation of 0.196, with a negative RE, reflecting significantly inferior performance.

For the PM_10 (Top 10% Prediction Accuracy) metric, at Tst = 0.15, GBLUP_Ad performs better with a mean of 30.000 compared to 20.000 for GBLUP. Both models have the same standard deviation of 25.820, indicating that GBLUP_Ad is superior in terms of prediction accuracy. As Tst increases, GBLUP_Ad continues to show better results. At Tst = 0.70, GBLUP_Ad has a mean of 34.545 and a standard deviation of 11.175, while GBLUP shows a mean of 12.727 and a similar standard deviation, highlighting the advantage of GBLUP_Ad.

Finally, for the PM_20 (Top 20% Prediction Accuracy) metric and for Tst = 0.15, GBLUP_Ad again outperforms GBLUP with a mean of 40.000 compared to 20.000. Although GBLUP_Ad has a higher standard deviation (21.082 vs. 15.811), its overall performance is superior. At Tst = 0.70, GBLUP_Ad maintains its advantage with a mean of 47.391 and a standard deviation of 8.056, while GBLUP has a mean of 20.435 and a slightly higher standard deviation, confirming the better performance of GBLUP_Ad with mean of 47.391.

### 3.4. Across Data

**Figure 4:**
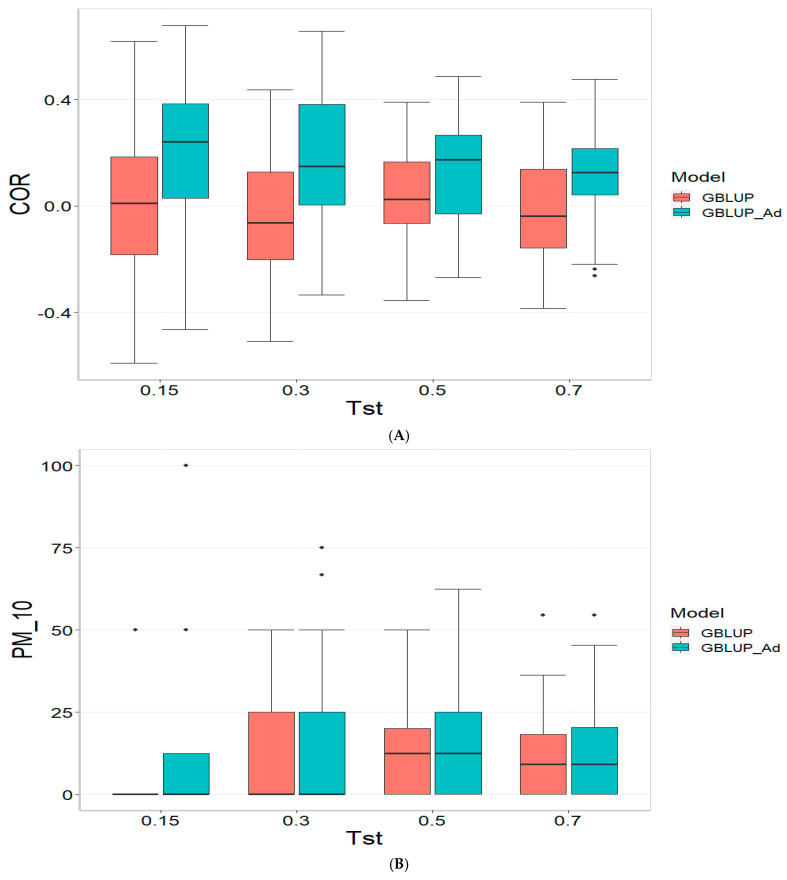

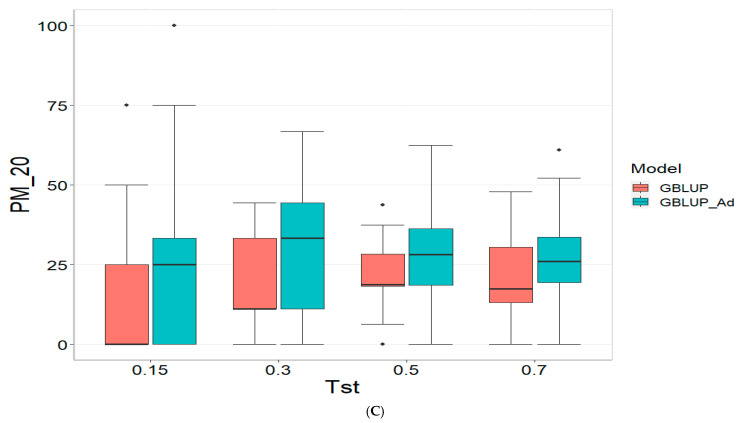
Comparative performance of genomic prediction models in terms of Pearson correlation (COR) (**A**), and percentage of agreement in the top 10% (PM_10) (**B**) and top 20% (PM_20) (**C**) for across data, using random cross-validation. Tst denotes the proportion of testing set. For each metric (COR, PM_10, PM_20), standard errors were calculated across the 10 cross-validation folds. These error bars provide an estimate of variability and aid in the interpretation of model stability across replicates.

Finally, the across data results are presented in Figure 4. For further details, please refer to Table A4 in Appendix A.

For the COR (Correlation) metric and for TST = 0.15, GBLUP shows a mean value close to zero (−0.001) and a standard deviation of 0.243, indicating high variability in predictions. Additionally, the relative efficiency (RE) is extremely negative (−16,136.276), suggesting very poor performance compared to GBLUP_Ad. As Tst increases, GBLUP continues to show low or negative mean values and higher standard deviations, indicating inconsistent predictions. For instance, at TST = 0.70, GBLUP has a mean of −0.004 and a standard deviation of 0.186, with a negative RE of −3316.083.

In the PM_10 (Top 10% Prediction Accuracy) and PM_20 (Top 20% Prediction Accuracy) metrics, GBLUP also demonstrates lower performance compared to GBLUP_Ad. For example, at TST = 0.15, GBLUP has a mean of 7.500 in PM_10 and 14.167 in PM_20, with relatively high standard deviations, indicating variability in predictions. In comparison, GBLUP_Ad has higher means in both metrics. As Tst increases, GBLUP continues to show lower means and considerable standard deviations. At TST = 0.70, GBLUP has a mean of 10.909 in PM_10 and 20.995 in PM_20, with standard deviations that indicate significant dispersion in the results compared to means of 13.030 and 26.415 for GBLUP_Ad for PM_10 and PM_20, respectively.

## 4. Discussion

Predicting the performance of tested lines in new environments poses significant challenges in genomic prediction due to the complexity of genotype-by-environment (G × E) interactions [13]. When moving to new environments, conditions such as climate, soil quality, and local agricultural practices may vary considerably, impacting the expression of genetic traits in ways that are often unpredictable from data in known environments [5]. This variability in environmental factors can interact with the genetic composition of a line, complicating the extrapolation of performance predictions [13].

Another major issue is the limited data on how different lines perform across diverse environments. Genomic prediction models rely on historical data, which often represents only a subset of possible conditions, limiting the models’ ability to generalize to new environments [1]. Moreover, these models are usually calibrated with specific environmental trials, making them highly tailored to those conditions. As a result, predictions in new settings may fail to accurately capture relevant environmental interactions, leading to reduced prediction accuracy [5,14].

Addressing these limitations often requires collecting extensive multi-environment trial data or developing sophisticated models that can better capture and adjust for G × E interactions. These approaches, however, involve significant resource investments, underscoring the ongoing challenge of predicting performance in new environments for genomic selection and plant breeding programs [14,15].

Our results show that across datasets, the proposed strategy of enriching the training set with data from other environments significantly outperforms the approach of using only target environment data. Gains observed in Pearson’s correlation were notable across all tested proportions of the testing set. For instance, with a testing proportion of 15%, 30%, 50% and 70%, the observed Pearson’s correlation gains were at least of 189.00%, 219.23%, 328.125%, and 2950%, respectively. Similarly, improvements in PM_10 were observed, with gains of 100% (in 15% testing), 69.84% (in 30% testing), 18.42% (in 50% testing), and 19.44% (in 70% testing), while PM_20 gains reached 82.35%, 61.83%, 20.79%, and 25.82%, respectively. These findings underscore the importance of incorporating data from additional environments into the training set. However, it is worth noting that despite the substantial relative gains, the absolute prediction accuracies achieved in these environments were generally below 0.5 in terms of Pearson’s correlation. This suggests a limited relationship between the environments used for enrichment and the target environment, India. This observation aligns with the fact that the enrichment environments included data from Obregon, Mexico, as well as from India in a previous year, and in some cases, from both locations combined.

These results underscore the potential of enriching target environments with information from other environments. However, the gains achieved are not uniform, which can be attributed to the significant heterogeneity among the environments used for enrichment. Consequently, it is recommended to prioritize enrichment using environments that closely resemble the target environment. Nonetheless, this approach is not always practical, as the number of available environments for enrichment may be limited, and they may not closely align with the target environment. Despite these challenges, the findings are generally promising, as they demonstrate that enriching target environments with data from similar environments can effectively enhance prediction performance.

These challenges are well-documented in the literature [14,15], and they underscore the need for models that can more effectively account for non-additive G × E patterns or integrate environmental covariables directly into prediction frameworks. For example, Taïbi et al. (2015) [16] demonstrated how phenotypic plasticity and local adaptation strongly influenced reforestation success in *Pinus halepensis*, underlining the critical role of G × E interaction and environmental fit in predictive performance. Our findings highlight the practical reality faced by breeders: even when model improvement is observed, absolute prediction accuracy may remain modest due to underlying biological complexity and environmental divergence between training and testing sets.

Finally, these results further strengthen the empirical evidence supporting the effectiveness of the GS methodology in uni-environment settings. When genetic material is relatively homogeneous and management practices are well-standardized, GS demonstrates a remarkable ability to deliver accurate predictions. This is particularly advantageous in controlled breeding programs where minimizing environmental variability is crucial for isolating genetic effects. The consistency of GS in such settings not only enhances prediction reliability but also supports more efficient selection decisions, ultimately accelerating genetic gain. Furthermore, these findings highlight the importance of carefully managing experimental conditions and selecting environments with minimal heterogeneity to maximize the utility of GS in practical applications [3,17].

### 4.1. Contrasting Sparse Testing Methodologies and Results from This Study, Montesinos et al. (2024) [8], and Burgueno et al. (2012) [7]

#### 4.1.1. Montesinos et al. (2024) [8]

This study explored genomic predictions under sparse conditions, employing both incomplete block design (IBD) and random allocation of genotypes to environments. Six GBLUP models were assessed, with one model (GBLUP_TRN) directly utilizing observed data without imputing missing values. The primary goal was to ascertain the benefits or disadvantages of pre-imputation versus the direct use of available genomic and phenotypic information. The practical advantages are no reliance on imputation, reduced computational complexity, and a realistic scenario for breeding programs with resource constraints. 

#### 4.1.2. This Research

In this study, the authors advanced the CV2 concept by assessing prediction strategies for tested genotypes in previously untested environments. The genomic prediction was implemented through two major approaches: training exclusively on the target environment data and training enriched by additional relevant environments, notably Obregon (Mexico) and historical Indian trials. Predictive accuracy was evaluated using correlations and the percentage of top-performing lines correctly identified (PM_10, PM_20), emphasizing practical implications in selection efficiency. Enhanced predictive accuracy through enriched training datasets and improved identification of high-performing genotypes in untested environments are some advantages, whereas disadvantages include dependency on the availability and relevance of external historical data and potential biases if external data differ significantly from target environments.

#### 4.1.3. Burgueño et al. (2012) [7]

This foundational study served as a benchmark for evaluating various statistical models’ robustness and predictive capabilities under realistically masked data. Advantages are the robust framework for evaluating model performance under realistic breeding conditions and the comprehensive modeling of G × E interactions; however, the method requires extensive computational resources for factorial analysis model implementation and may be overly complex for small-scale or less-resourced breeding programs.

Collectively, Table 2 shows that the results from [7], this study, and [6] underscore the critical role CV2 validation plays in realistically assessing genomic prediction models in plant breeding. Each study uniquely contributes to the methodological refinement and application of CV2 schemes, demonstrating different advantages: direct genomic prediction from sparse testing conditions [7], leveraging enriched datasets to enhance accuracy in untested environments (this study), and comprehensive model comparison under structured masking conditions [6].

Overall, the strategic use of CV2 validations, combined with methodological adaptations tailored to practical breeding scenarios and the integration of environmental covariables, highlights a powerful pathway toward more accurate and resource-efficient genomic selection in plant breeding programs.

### 4.2. Factors Limiting Prediction Accuracy Across Environments

Despite the consistent performance improvement of GBLUP_Ad over GBLUP, we observed that the overall Pearson’s correlation values remained below 0.5 in many cases. This is not unexpected in multi-environment genomic prediction involving sparse testing across heterogeneous environments. One major factor limiting predictive accuracy is the presence of strong genotype-by-environment (G × E) interactions, where the expression of genetic effects varies with environmental context. The contrasting environmental conditions and agronomic management practices between the Indian test sites and Obregon (Mexico) likely contribute to non-transferable genotype performance, especially for yield-related traits that are highly sensitive to local stresses. These challenges are well-documented in the literature [13,14]; for example, Taïbi et al. (2015) [16] demonstrated how phenotypic plasticity and local adaptation strongly influenced reforestation success in *Pinus halepensis*, underlining the critical role of G × E interaction and environmental fit in predictive performance. Our findings highlight the practical reality faced by breeders: even when model improvement is observed, absolute prediction accuracy may remain modest due to underlying biological complexity and environmental divergence between training and testing sets.

## 5. Conclusions

From our results, we conclude that utilizing data from diverse environments can significantly enhance prediction accuracy in new environments with sparse testing. By integrating information from multiple environmental contexts, genomic prediction models can capture a broader range of genotype-by-environment (G × E) interactions, thereby improving their ability to generalize to unfamiliar conditions. This approach allows models to more accurately estimate genetic responses under varying environmental pressures, increasing their robustness and reliability in settings with limited testing data. While challenges in data collection and model complexity remain, leveraging multi-environment data offers a promising strategy to overcome the limitations of sparse testing, facilitating better decision making in plant breeding and selection. However, even with improved prediction accuracy through data from diverse environments, the overall accuracy remains relatively low. This limitation arises because G × E interactions are highly complex and often specific to environmental conditions, which are challenging to fully capture and generalize. While multi-environmental data enrich the model, they cannot account for all potential environmental variables or their interactions with genotypes in every new setting. Thus, despite gains from this approach, prediction accuracies in new environments remain constrained by the inherent variability and unpredictable nature of G × E interactions, underscoring the need for continuous model refinement and advanced strategies to enhance prediction reliability in plant breeding.

## Figures and Tables

**Table 1:** Description of the wheat datasets. MAF denotes the minor allele frequency and PMV denotes the threshold of percentage of missing values.

No.	Data	Lines	Markers	Env_India	Env_Mexico	MAF	PMV
1	TPE_1_2021_2022	166	18,238	4	3	0.05	50%
2	TPE_1_2022_2023	166	18,238	6	6	0.05	50%
3	TPE_2_2021_2022	166	18,238	5	3	0.05	50%
4	TPE_2_2022_2023	165	18,238	6	6	0.05	50%
5	TPE_3_2021_2022	112	18,238	2	3	0.05	50%
6	TPE_3_2022_2023	166	18,238	3	6	0.05	50%

**Table 2:** Comparative summary of methodologies.

Feature	Montesinos et al. (2024) [8]	This Study	Burgueño et al. (2012) [7]
Crop	Wheat	Wheat	Wheat
Cross-Validation Scheme	CV2	CV2	CV2
Data Design	Sparse testing: IBD and Random	Sparse testing: targeted enrichment	Systematic random masking
Genotype–Environment Coverage	All genotypes observed at least once	Some genotypes entirely unobserved	Balanced masking across environments
Prediction Models	GBLUP (multiple variants)	GBLUP enriched with external datasets	Pedigree, markers, FA structures
Modeling G × E Interaction	Yes (covariance structure)	Yes (multi-environment integration)	Yes (FA models explicitly modeling covariance)
Evaluation Metrics	COR, NRMSE, PM_10, PM_20	COR, PM_10, PM_20	COR

**Figure A1:**
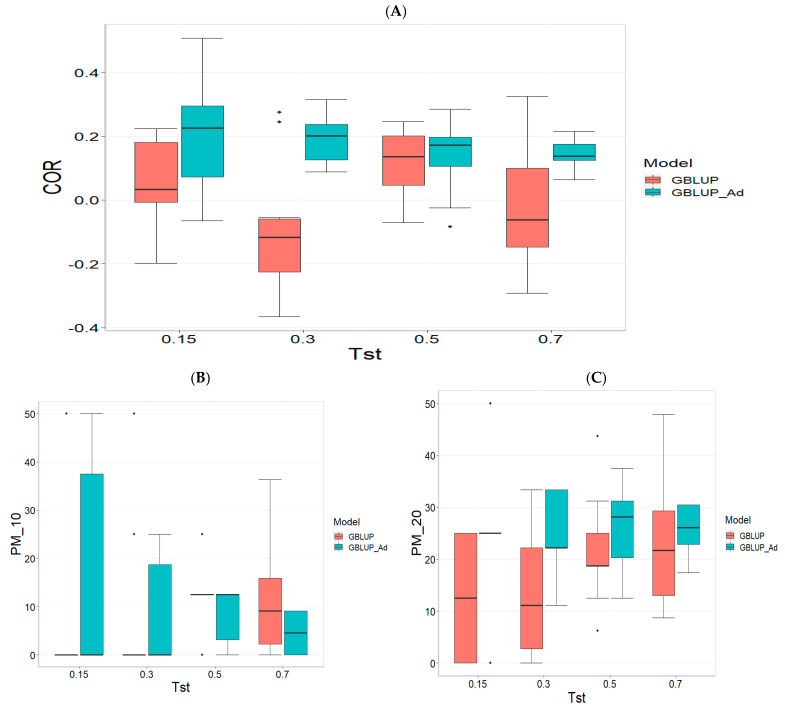
Comparative performance of genomic prediction models in terms of Pearson correlation (COR) (**A**), and percentage of agreement in the top 10% (PM_10) (**B**) and top 20% (PM_20) (**C**) for TPE_1_2022_2023, using random cross-validation. Tst denotes the proportion of testing set. For each metric (COR, PM_10, PM_20), standard errors were calculated across the 10 cross-validation folds. These error bars provide an estimate of variability and aid in the interpretation of model stability across replicates.

**Figure A2:**
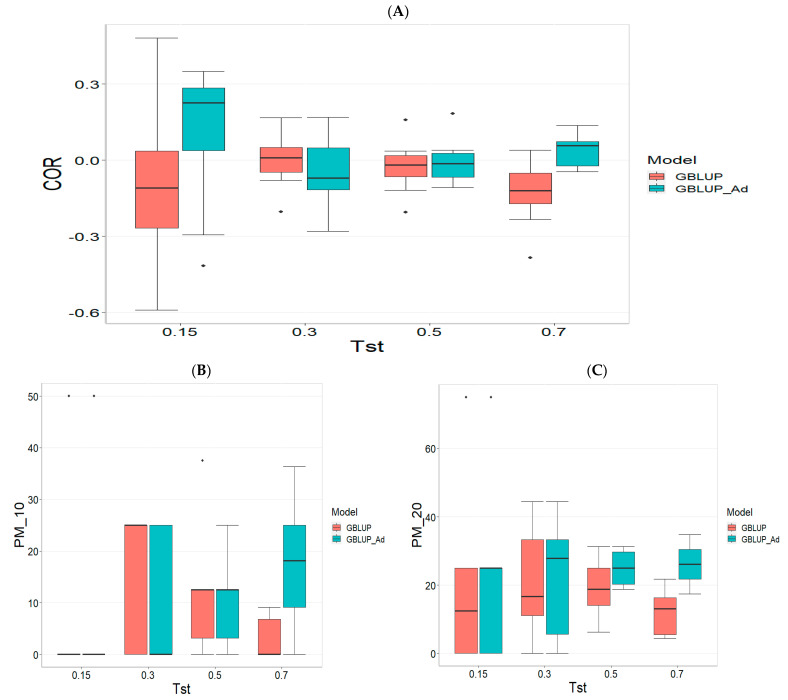
Comparative performance of genomic prediction models in terms of Pearson correlation (COR) (**A**), and percentage of agreement in the top 10% (PM_10) (**B**) and top 20% (PM_20) (**C**) for TPE_2_2022_2023, using random cross-validation. Tst denotes the proportion of testing set. For each metric (COR, PM_10, PM_20), standard errors were calculated across the 10 cross-validation folds. These error bars provide an estimate of variability and aid in the interpretation of model stability across replicates.

**Figure A3:**
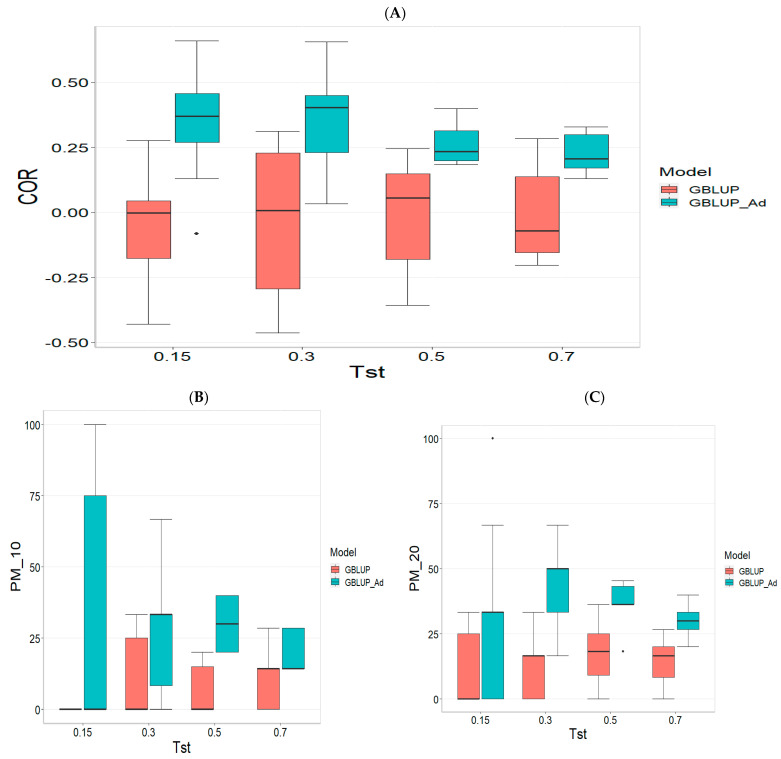
Comparative performance of genomic prediction models in terms of Pearson correlation (COR) (**A**), and percentage of agreement in the top 10% (PM_10) (**B**) and top 20% (PM_20) (**C**) for TPE_3_2021_2022, using random cross-validation. Tst denotes the proportion of testing set. For each metric (COR, PM_10, PM_20), standard errors were calculated across the 10 cross-validation folds. These error bars provide an estimate of variability and aid in the interpretation of model stability across replicates.

## Data Availability

All phenotypic data, genotype marker matrices, R scripts, and parameter settings used in this study are fully available at the following GitHub repository: https://github.com/osval78/Sparse_testing_Across (accessed on 28 July 2024). The repository includes scripts for data preprocessing, model fitting using the BGLR package [10], and performance evaluation across cross-validation scenarios. A detailed README file provides instructions for reproducing the analyses presented in this manuscript.

## Appendix A

**Table A1:** Comparative performance of genomic prediction models in terms of Pearson’s correlation (COR) (A), and Percentage of Matching in top 10% (PM_10) (B) and top 20% (PM_20) (C) for the TPE_1_2021_2022 dataset under random cross-validation. Tst denotes the proportion of testing set.

Metric	Model	Tst	Min	Mean	Max	Sd	RE (%)
COR	GBLUP	0.15	−0.390	−0.017	0.618	0.312	−1156.801
COR	GBLUP_Ad	0.15	−0.172	0.179	0.439	0.180	0.000
COR	GBLUP	0.30	−0.322	−0.045	0.262	0.177	−402.346
COR	GBLUP_Ad	0.30	−0.049	0.137	0.344	0.113	0.000
COR	GBLUP	0.50	−0.218	0.103	0.390	0.184	45.852
COR	GBLUP_Ad	0.50	0.033	0.150	0.245	0.064	0.000
COR	GBLUP	0.70	−0.192	0.091	0.391	0.207	10.133
COR	GBLUP_Ad	0.70	0.028	0.101	0.177	0.047	0.000
PM_10	GBLUP	0.15	0.000	5.000	50.000	15.811	0.000
PM_10	GBLUP_Ad	0.15	0.000	5.000	50.000	15.811	0.000
PM_10	GBLUP	0.30	0.000	7.500	25.000	12.076	−66.667
PM_10	GBLUP_Ad	0.30	0.000	2.500	25.000	7.906	0.000
PM_10	GBLUP	0.50	0.000	13.750	50.000	18.114	−63.636
PM_10	GBLUP_Ad	0.50	0.000	5.000	25.000	8.740	0.000
PM_10	GBLUP	0.70	9.091	15.455	27.273	7.484	−88.235
PM_10	GBLUP_Ad	0.70	0.000	1.818	18.182	5.750	0.000
PM_20	GBLUP	0.15	0.000	7.500	50.000	16.874	233.333
PM_20	GBLUP_Ad	0.15	0.000	25.000	75.000	20.412	0.000
PM_20	GBLUP	0.30	0.000	17.778	44.444	14.055	56.250
PM_20	GBLUP_Ad	0.30	11.111	27.778	44.444	14.103	0.000
PM_20	GBLUP	0.50	6.250	24.375	43.750	11.200	−5.128
PM_20	GBLUP_Ad	0.50	12.500	23.125	31.250	7.247	0.000
PM_20	GBLUP	0.70	8.696	27.391	43.478	12.971	−12.698
PM_20	GBLUP_Ad	0.70	17.391	23.913	34.783	5.519	0.000

**Table A2:** Comparative performance of genomic prediction models in terms of Pearson’s correlation (COR) (A), and Percentage of Matching in top 10% (PM_10) (B) and top 20% (PM_20) (C) for the TPE_2_2021_2022 dataset under random cross-validation. Tst denotes the proportion of testing set.

Metric	Model	Tst	Min	Mean	Max	Sd	RE (%)
COR	GBLUP	0.15	−0.419	0.024	0.343	0.234	−718.437
COR	GBLUP_Ad	0.15	−0.464	−0.148	0.135	0.212	0.000
COR	GBLUP	0.30	−0.510	−0.166	0.024	0.181	−21.570
COR	GBLUP_Ad	0.30	−0.335	−0.130	0.046	0.124	0.000
COR	GBLUP	0.50	−0.200	−0.016	0.177	0.115	809.800
COR	GBLUP_Ad	0.50	−0.271	−0.148	−0.087	0.057	0.000
COR	GBLUP	0.70	−0.181	0.081	0.361	0.159	−340.741
COR	GBLUP_Ad	0.70	−0.264	−0.194	−0.107	0.046	0.000
PM_10	GBLUP	0.15	0.000	5.000	50.000	15.811	−100.000
PM_10	GBLUP_Ad	0.15	0.000	0.000	0.000	0.000	NA
PM_10	GBLUP	0.30	0.000	7.500	25.000	12.076	−100.000
PM_10	GBLUP_Ad	0.30	0.000	0.000	0.000	0.000	NA
PM_10	GBLUP	0.50	0.000	12.500	25.000	8.333	−90.000
PM_10	GBLUP_Ad	0.50	0.000	1.250	12.500	3.953	0.000
PM_10	GBLUP	0.70	0.000	13.636	54.545	16.177	−100.000
PM_10	GBLUP_Ad	0.70	0.000	0.000	0.000	0.000	NA
PM_20	GBLUP	0.15	0.000	17.500	50.000	20.582	−57.143
PM_20	GBLUP_Ad	0.15	0.000	7.500	50.000	16.874	0.000
PM_20	GBLUP	0.30	0.000	16.667	44.444	14.103	−80.000
PM_20	GBLUP_Ad	0.30	0.000	3.333	22.222	7.499	0.000
PM_20	GBLUP	0.50	0.000	21.250	37.500	12.569	−76.471
PM_20	GBLUP_Ad	0.50	0.000	5.000	12.500	3.953	0.000
PM_20	GBLUP	0.70	13.043	28.696	47.826	12.332	−84.848
PM_20	GBLUP_Ad	0.70	0.000	4.348	8.696	2.899	0.000

**Table A3:** Comparative performance of genomic prediction models in terms of Pearson’s correlation (COR) (A), and Percentage of Matching in top 10% (PM_10) (B) and top 20% (PM_20) (C) for the TPE_3_2022_2023 dataset under random cross-validation. Tst denotes the proportion of testing set.

Metric	Model	Tst	Min	Mean	Max	Sd	RE (%)
COR	GBLUP	0.15	−0.366	0.073	0.364	0.236	519.809
COR	GBLUP_Ad	0.15	0.335	0.455	0.677	0.104	0.000
COR	GBLUP	0.30	−0.404	0.018	0.436	0.263	2501.594
COR	GBLUP_Ad	0.30	0.378	0.481	0.640	0.072	0.000
COR	GBLUP	0.50	−0.285	0.031	0.285	0.193	1284.182
COR	GBLUP_Ad	0.50	0.336	0.425	0.486	0.044	0.000
COR	GBLUP	0.70	−0.366	−0.029	0.274	0.196	−1522.158
COR	GBLUP_Ad	0.70	0.372	0.418	0.476	0.029	0.000
PM_10	GBLUP	0.15	0.000	20.000	50.000	25.820	50.000
PM_10	GBLUP_Ad	0.15	0.000	30.000	50.000	25.820	0.000
PM_10	GBLUP	0.30	0.000	5.000	25.000	10.541	750.000
PM_10	GBLUP_Ad	0.30	25.000	42.500	75.000	16.874	0.000
PM_10	GBLUP	0.50	0.000	17.500	37.500	12.076	92.857
PM_10	GBLUP_Ad	0.50	12.500	33.750	62.500	14.494	0.000
PM_10	GBLUP	0.70	0.000	12.727	27.273	10.671	171.429
PM_10	GBLUP_Ad	0.70	18.182	34.545	54.545	11.175	0.000
PM_20	GBLUP	0.15	0.000	20.000	50.000	15.811	100.000
PM_20	GBLUP_Ad	0.15	0.000	40.000	75.000	21.082	0.000
PM_20	GBLUP	0.30	0.000	20.000	44.444	17.213	122.222
PM_20	GBLUP_Ad	0.30	33.333	44.444	66.667	9.072	0.000
PM_20	GBLUP	0.50	18.750	28.125	43.750	9.433	55.556
PM_20	GBLUP_Ad	0.50	31.250	43.750	62.500	10.206	0.000
PM_20	GBLUP	0.70	4.348	20.435	30.435	8.946	131.915
PM_20	GBLUP_Ad	0.70	39.130	47.391	60.870	8.056	0.000

**Table A4:** Comparative performance of genomic prediction models in terms of Pearson’s correlation (COR) (A), and Percentage of Matching in top 10% (PM_10) (B) and top 20% (PM_20) (C) for the across data under random cross-validation. Tst denotes the proportion of testing set.

Metric	Model	Tst	Min	Mean	Max	Sd	RE (%)
COR	GBLUP	0.15	−0.591	−0.001	0.618	0.243	18900
COR	GBLUP_Ad	0.15	−0.464	0.190	0.677	0.271	0.000
COR	GBLUP	0.30	−0.510	−0.052	0.436	0.214	219.23
COR	GBLUP_Ad	0.30	−0.335	0.166	0.655	0.245	0.000
COR	GBLUP	0.50	−0.357	0.032	0.390	0.165	328.125
COR	GBLUP_Ad	0.50	−0.271	0.137	0.486	0.199	0.000
COR	GBLUP	0.70	−0.385	−0.004	0.391	0.186	2950
COR	GBLUP_Ad	0.70	−0.264	0.122	0.476	0.194	0.000
PM_10	GBLUP	0.15	0.000	7.500	50.000	18.004	100.000
PM_10	GBLUP_Ad	0.15	0.000	15.000	100.000	28.074	0.000
PM_10	GBLUP	0.30	0.000	8.750	50.000	13.413	69.841
PM_10	GBLUP_Ad	0.30	0.000	14.861	75.000	19.951	0.000
PM_10	GBLUP	0.50	0.000	12.667	50.000	12.186	18.421
PM_10	GBLUP_Ad	0.50	0.000	15.000	62.500	15.404	0.000
PM_10	GBLUP	0.70	0.000	10.909	54.545	10.900	19.444
PM_10	GBLUP_Ad	0.70	0.000	13.030	54.545	14.353	0.000
PM_20	GBLUP	0.15	0.000	14.167	75.000	17.847	82.353
PM_20	GBLUP_Ad	0.15	0.000	25.833	100.000	24.390	0.000
PM_20	GBLUP	0.30	0.000	17.222	44.444	14.014	61.828
PM_20	GBLUP_Ad	0.30	0.000	27.870	66.667	18.679	0.000
PM_20	GBLUP	0.50	0.000	22.045	43.750	10.769	20.790
PM_20	GBLUP_Ad	0.50	0.000	26.629	62.500	14.212	0.000
PM_20	GBLUP	0.70	0.000	20.995	47.826	11.963	25.817
PM_20	GBLUP_Ad	0.70	0.000	26.415	60.870	13.894	0.000

## Appendix C

**Table A5:** Comparative performance of genomic prediction models in terms of Pearson’s correlation (COR) (A), and Percentage of Matching in top 10% (PM_10) (B) and top 20% (PM_20) (C) for TPE_1_2022_2023, TPE_2_2022_2023 and TPE_3_2021_2022 datasets under random cross-validation. Tst denotes the proportion of testing set.

Data	Metric	Model	Tst	Min	Mean	Max	Sd	RE (%)
TPE_1_2022_2023	COR	GBLUP	0.15	−0.20	0.06	0.22	0.14	224.83
TPE_1_2022_2023	COR	GBLUP_Ad	0.15	−0.07	0.20	0.51	0.18	0.00
TPE_1_2022_2023	COR	GBLUP	0.30	−0.37	−0.09	0.27	0.21	−302.93
TPE_1_2022_2023	COR	GBLUP_Ad	0.30	0.09	0.19	0.32	0.07	0.00
TPE_1_2022_2023	COR	GBLUP	0.50	−0.07	0.12	0.25	0.11	15.11
TPE_1_2022_2023	COR	GBLUP_Ad	0.50	−0.08	0.14	0.29	0.12	0.00
TPE_1_2022_2023	COR	GBLUP	0.70	−0.29	−0.03	0.33	0.19	−620.93
TPE_1_2022_2023	COR	GBLUP_Ad	0.70	0.06	0.15	0.22	0.05	0.00
TPE_1_2022_2023	PM_10	GBLUP	0.15	0.00	10.00	50.00	21.08	50.00
TPE_1_2022_2023	PM_10	GBLUP_Ad	0.15	0.00	15.00	50.00	24.15	0.00
TPE_1_2022_2023	PM_10	GBLUP	0.30	0.00	7.50	50.00	16.87	0.00
TPE_1_2022_2023	PM_10	GBLUP_Ad	0.30	0.00	7.50	25.00	12.08	0.00
TPE_1_2022_2023	PM_10	GBLUP	0.50	0.00	12.50	25.00	8.33	−30.00
TPE_1_2022_2023	PM_10	GBLUP_Ad	0.50	0.00	8.75	12.50	6.04	0.00
TPE_1_2022_2023	PM_10	GBLUP	0.70	0.00	10.91	36.36	11.18	−58.33
TPE_1_2022_2023	PM_10	GBLUP_Ad	0.70	0.00	4.55	9.09	4.79	0.00
TPE_1_2022_2023	PM_20	GBLUP	0.15	0.00	12.50	25.00	13.18	120.00
TPE_1_2022_2023	PM_20	GBLUP_Ad	0.15	0.00	27.50	50.00	14.19	0.00
TPE_1_2022_2023	PM_20	GBLUP	0.30	0.00	14.44	33.33	12.88	69.23
TPE_1_2022_2023	PM_20	GBLUP_Ad	0.30	11.11	24.44	33.33	8.76	0.00
TPE_1_2022_2023	PM_20	GBLUP	0.50	6.25	21.88	43.75	10.31	17.14
TPE_1_2022_2023	PM_20	GBLUP_Ad	0.50	12.50	25.63	37.50	8.56	0.00
TPE_1_2022_2023	PM_20	GBLUP	0.70	8.70	23.04	47.83	13.29	11.32
TPE_1_2022_2023	PM_20	GBLUP_Ad	0.70	17.39	25.65	30.43	5.21	0.00
TPE_2_2022_2023	COR	GBLUP	0.15	−0.59	−0.09	0.48	0.31	−225.53
TPE_2_2022_2023	COR	GBLUP_Ad	0.15	−0.42	0.11	0.35	0.27	0.00
TPE_2_2022_2023	COR	GBLUP	0.30	−0.20	0.01	0.17	0.11	−659.51
TPE_2_2022_2023	COR	GBLUP_Ad	0.30	−0.28	−0.04	0.17	0.14	0.00
TPE_2_2022_2023	COR	GBLUP	0.50	−0.21	−0.03	0.16	0.10	−75.61
TPE_2_2022_2023	COR	GBLUP_Ad	0.50	−0.11	−0.01	0.18	0.08	0.00
TPE_2_2022_2023	COR	GBLUP	0.70	−0.39	−0.12	0.04	0.13	−130.06
TPE_2_2022_2023	COR	GBLUP_Ad	0.70	−0.05	0.04	0.14	0.06	0.00
TPE_2_2022_2023	PM_10	GBLUP	0.15	0.00	5.00	50.00	15.81	100.00
TPE_2_2022_2023	PM_10	GBLUP_Ad	0.15	0.00	10.00	50.00	21.08	0.00
TPE_2_2022_2023	PM_10	GBLUP	0.30	0.00	15.00	25.00	12.91	−33.33
TPE_2_2022_2023	PM_10	GBLUP_Ad	0.30	0.00	10.00	25.00	12.91	0.00
TPE_2_2022_2023	PM_10	GBLUP	0.50	0.00	13.75	37.50	13.76	−18.18
TPE_2_2022_2023	PM_10	GBLUP_Ad	0.50	0.00	11.25	25.00	9.22	0.00
TPE_2_2022_2023	PM_10	GBLUP	0.70	0.00	2.73	9.09	4.39	533.33
TPE_2_2022_2023	PM_10	GBLUP_Ad	0.70	0.00	17.27	36.36	10.88	0.00
TPE_2_2022_2023	PM_20	GBLUP	0.15	0.00	17.50	75.00	23.72	42.86
TPE_2_2022_2023	PM_20	GBLUP_Ad	0.15	0.00	25.00	75.00	28.87	0.00
TPE_2_2022_2023	PM_20	GBLUP	0.30	0.00	21.11	44.44	14.30	5.26
TPE_2_2022_2023	PM_20	GBLUP_Ad	0.30	0.00	22.22	44.44	16.56	0.00
TPE_2_2022_2023	PM_20	GBLUP	0.50	6.25	19.38	31.25	9.06	29.03
TPE_2_2022_2023	PM_20	GBLUP_Ad	0.50	18.75	25.00	31.25	5.10	0.00
TPE_2_2022_2023	PM_20	GBLUP	0.70	4.35	11.74	21.74	6.17	125.93
TPE_2_2022_2023	PM_20	GBLUP_Ad	0.70	17.39	26.52	34.78	5.96	0.00
TPE_3_2021_2022	COR	GBLUP	0.15	−0.43	−0.06	0.28	0.20	−640.49
TPE_3_2021_2022	COR	GBLUP_Ad	0.15	−0.08	0.35	0.66	0.22	0.00
TPE_3_2021_2022	COR	GBLUP	0.30	−0.46	−0.03	0.31	0.29	−1220.66
TPE_3_2021_2022	COR	GBLUP_Ad	0.30	0.03	0.36	0.66	0.19	0.00
TPE_3_2021_2022	COR	GBLUP	0.50	−0.36	−0.02	0.24	0.22	−1508.08
TPE_3_2021_2022	COR	GBLUP_Ad	0.50	0.18	0.26	0.40	0.08	0.00
TPE_3_2021_2022	COR	GBLUP	0.70	−0.20	−0.01	0.28	0.17	−1825.50
TPE_3_2021_2022	COR	GBLUP_Ad	0.70	0.13	0.22	0.33	0.07	0.00
TPE_3_2021_2022	PM_10	GBLUP	0.15	0.00	0.00	0.00	0.00	Inf
TPE_3_2021_2022	PM_10	GBLUP_Ad	0.15	0.00	30.00	100.00	48.30	0.00
TPE_3_2021_2022	PM_10	GBLUP	0.30	0.00	10.00	33.33	16.10	166.67
TPE_3_2021_2022	PM_10	GBLUP_Ad	0.30	0.00	26.67	66.67	21.08	0.00
TPE_3_2021_2022	PM_10	GBLUP	0.50	0.00	6.00	20.00	9.66	400.00
TPE_3_2021_2022	PM_10	GBLUP_Ad	0.50	20.00	30.00	40.00	10.54	0.00
TPE_3_2021_2022	PM_10	GBLUP	0.70	0.00	10.00	28.57	9.64	100.00
TPE_3_2021_2022	PM_10	GBLUP_Ad	0.70	14.29	20.00	28.57	7.38	0.00
TPE_3_2021_2022	PM_20	GBLUP	0.15	0.00	10.00	33.33	16.10	200.00
TPE_3_2021_2022	PM_20	GBLUP_Ad	0.15	0.00	30.00	100.00	33.15	0.00
TPE_3_2021_2022	PM_20	GBLUP	0.30	0.00	13.33	33.33	13.15	237.50
TPE_3_2021_2022	PM_20	GBLUP_Ad	0.30	16.67	45.00	66.67	15.81	0.00
TPE_3_2021_2022	PM_20	GBLUP	0.50	0.00	17.27	36.36	10.88	115.79
TPE_3_2021_2022	PM_20	GBLUP_Ad	0.50	18.18	37.27	45.45	7.96	0.00
TPE_3_2021_2022	PM_20	GBLUP	0.70	0.00	14.67	26.67	8.20	109.09
TPE_3_2021_2022	PM_20	GBLUP_Ad	0.70	20.00	30.67	40.00	6.44	0.00
